# miR-328 mediates a metabolic shift in colon cancer cells by targeting SLC2A1/GLUT1

**DOI:** 10.1007/s12094-018-1836-1

**Published:** 2018-01-26

**Authors:** S. Santasusagna, I. Moreno, A. Navarro, C. Muñoz, F. Martinez, R. Hernández, J. J. Castellano, M. Monzo

**Affiliations:** 1 Molecular Oncology and Embryology Laboratory, Human Anatomy Unit, School of Medicine, University of Barcelona, IDIBAPS, Barcelona, Spain; 2 Department of Medical Oncology and Surgery, Hospital Municipal de Badalona, Badalona, Spain; 3 Unit of Human Anatomy and Embryology, School of Medicine, Hospital Clinic, University of Barcelona, Casanova 143, 08036 Barcelona, Spain

**Keywords:** SLC2A1, GLUT1, miR-328, Colon cancer, Glycolysis

## Abstract

**Purpose:**

Increasing evidence shows that altered metabolism is a critical hallmark in colon cancer. There is a strong need to explore the molecular mechanisms underlying cancer metabolism. Whether the aberrant expression of microRNAs contributes to cancer metabolism is not fully understood. miR-328 is a putative potential target of SLC2A1, but the regulating mechanism between them remains unknown. We have examined whether miR-328 directly regulates SLC2A1/GLUT1 expression in colon cancer cells.

**Methods:**

We performed in silico bioinformatic analyses to identify miR-328-mediated molecular pathways and targets. We also performed luciferase assays and western blot analyses in LOVO and SW480 colon cancer cell lines. In addition, we assessed miR-328 expression in 47 paired tumor and normal tissue specimens from resected colon cancer patients.

**Results:**

Luciferase reporter assays showed that miR-328 directly targeted SLC2A1 3′-untranslated region (UTR), with a significant decrease in luciferase activity in both LOVO and SW480 cell lines. These results were validated by western blot. miR-328 expression was significantly downregulated in tumor tissue compared with paired normal tissue.

**Conclusions:**

Our results show that miR-328 targets SLC2A1/GLUT1. We suggest that miR-328 may be involved in the orchestration of the Warburg effect in colon cancer cells. Furthermore, miR‐328 expression is reduced in colon cancer patients and thus inversely correlates with the classically reported upregulated SLC2A1/GLUT1 expression in tumors.

## Introduction

Colorectal cancer is the third most common cancer worldwide in both males and females and represents the second and the third cause of cancer death in males and females, respectively [1], making it a significant health burden. The vast majority of colorectal cancer cases are sporadic and usually progress from a benign polyp to a malignant adenocarcinoma while cells accumulate a series of genetic and epigenetic changes [2]. For example, mutations in KRAS predict a failure to respond to EGFR inhibitors [3].

It is well known that the metabolism of cancer cells is significantly different from that of normally differentiated cells [4]. Cancer cells preferentially use aerobic glycolysis to metabolize glucose, which is a less efficient pathway than mitochondrial oxidative phosphorylation, the main metabolic pathway used by normal cells. This different metabolism, termed the Warburg effect, is characterized by increased glycolysis and lactate production [5], which is one of the main hallmarks of cancer and could be the result of adaptations designed to maintain the continuous proliferation of cancer cells. In this malignant scenario, one of the earliest mechanisms that is upregulated during oncogenesis is the uniport protein glucose transporter 1 (GLUT1), which is a pivotal rate-limiting element in the transport of glucose in cancer cells and is responsible for increasing their glucose uptake [6]. GLUT1 deregulation results in an increased glucose uptake into the cytoplasm of tumor cells [7]. GLUT1 has found to be overexpressed in various cancers, such as esophageal squamous cell carcinoma [8], gastric carcinoma [9] and colon cancer (CC) [10, 11], and is significantly associated with worse prognosis [11]. In addition, a recent meta-analysis found that GLUT1 expression could be a promising prognostic and therapeutic target in solid tumors [6].

Recently, a number of microRNAs (miRNAs) were identified as important natural regulators of metabolism [12, 13]. For example, in renal cell carcinoma, miR-1291 induces cell proliferation, migration and invasion by targeting solute career family 2 member 1 (SLC2A1), which encodes for GLUT1 [14]. In addition, miR-124 inhibits proliferation, glycolysis, and energy metabolism, by targeting AKT-GLUT1/HKII in non-small cell lung cancer cells [15]. Several studies have reported that miR-328 is dysregulated in numerous human cancers, thus compromising metabolic and non-metabolic pathways [16–21]. In a previous study by our group, miR-328 was present in exosomes isolated from the tumor-draining vein of CC patients and its expression was associated with liver metastases [22], leading us to speculate that miR-328 could play a role in the disruption of the metabolic pathway regulated by GLUT1 by directly binding to its 3′‐UTR. However, the direct function of miR‐328 in CC cells has not been examined. In the present study, we have analyzed the expression levels of miR-328 in a cohort of surgical CC patients to evaluate the relationship between miR-328 expression and its potential target SLC2A1.

## Methods

### Cell culture

Two human CC cell lines, LOVO (ATCC^®^ CCL-229^™^) and SW480 (ATCC^®^ CCL-228^™^), were provided by American Type Culture Collection (Manassas, USA). Both cell lines were cultured in Roswell Park Memorial Institute (RPMI) 1640 medium (Life Technologies, Carlsbad, CA, USA) with 10% fetal bovine serum (Invitrogen, Carlsbad, CA, USA) and 1% penicillin/streptomycin (Sigma-Aldrich, St. Louis, MO, USA) at 37 °C with 5% CO_2_.

### miRNA target prediction

Bioinformatics prediction of target genes and miRNA binding sites was performed using a combination of TargetScan version 5.1 (http://www.targetscan.com), miRanda August 2010 release (http://www.microrna.org), mirDB April 2009 (www.mirdb.org) and Tarbase (http://diana.imis.athena-innovation.gr/DianaTools/index.php?r=tarbase/index). To obtain a reliable miRNA target prediction, we used the algorithm mirSVR for scoring and ranking the efficiency of miRNA target sites, including both target site information and contextual features [23]. In addition, phastCons score was used to study the target site conservation of nucleotide positions across multiple vertebrates [24].

### miRNA transfection

One day before transfection, LOVO (8 × 10^5^) and SW480 (5 × 10^5^) cells were seeded in 6-well plates in RPMI media (Life Technologies) overnight. The following day, media were removed and cells were transfected with 50 ng of the pMirTarget vector (Origene), and 100 nM of the rellevant pre-miRNA or pre-miR-Negative Control#1 (Life Technologies) using Lipofectamine 2000 in Opti-mem media (Life Technologies) according to the manufacturer’s protocol. After 6 h transfection, the transfection reagent was replaced with complete RPMI media (Life Technologies). Cells were harvested for other experiments at 24 h after transfection. Transfection efficiency was monitored by qPCR.

### Luciferase assay

To validate the potential target genes identified, a Luciferase assay was performed in LOVO and SW480 cells using a modified vector with the 3′UTR region of SLC2A1 gene (pMirTarget-SLC2A1-3′-UTR; accession number: 006516) obtained from Origene Technologies Inc. (Rockville, MD, USA). The Luciferase activity was measured 24 h after transfection with the Promega Dual Luciferase Reporter Assay System (Promega, Madison, WI, USA) in a luminometer Versa Max Microplate Reader. Each experiment was performed at least three times. The effect of the miR-328 in the Luciferase assay was then confirmed by western blot.

### Western blot

Western blot analysis was performed in LOVO and SW480 cells using the following primary antibodies: GLUT1 (21829-1-AP, Proteintech) and α-tubulin (T 6074, Sigma-Aldrich). Briefly, the cell pellet was mixed with LDS sample buffer (Life Technologies) and sample reducing agent (Life Technologies), according to the manufacturer’s protocol. Proteins extracted were loaded onto a SDS-PAGE (Novex 4–12% Bis–Tris gel, Life Technologies), transferred to a nitrocellulose membrane by iBlot (Life Technologies), and blocked 1 h with 1× TBST buffer (Fisher Scientific) with 5% w/v nonfat dry milk. The membranes were incubated in blocking buffer at 4 °C overnight with primary antibody against GLUT1 (1:1000) or 1 h at room temperature for α-tubulin (1:3000). Subsequently, the membranes were washed three times with Tris-buffered saline with Tween-20 (TBST) and incubated 1 h with secondary antibodies (1:5000; anti-rabbit sc-2004, Santa Cruz Biotechnology; or 1:3000; anti-mouse A9044, Sigma-Aldrich). The signal was obtained using the Novex ECL Chemiluminescent Substrate Reagent kit (WP 20005, Invitrogen) and the images were developed and quantified using the Chemidoc System (Bio-Rad).

### Patient samples

From August 2009 to August 2013, samples were obtained from 47 patients with stage I to III CC who underwent surgical resection at the Municipal Hospital of Badalona. All 47 patients underwent a complete history and physical examination.

Approval for the study was obtained from the institutional review board of the hospital, and signed informed consent was obtained from all patients and controls in accordance with the Declaration of Helsinki.

For all 47 patients, we obtained tumor tissue and paired normal tissue. Normal tissue was obtained from the area of the colon farthest from the tumor. Both tumor and normal tissue samples were analyzed and confirmed by a pathologist and frozen at − 80 °C for further use.

### RNA extraction and miRNA quantification

Total RNA was extracted from fresh tumor and paired normal tissue using Trizol total RNA isolation reagent (Invitrogen) according to the manufacturer’s protocol. miRNA detection was performed using commercial assays (TaqMan MicroRNA assays, Life Technologies) for miR-328 (assay ID: 000543, Life Technologies), in the 7500 Sequence Detection System (Life Technologies). The appropriate negative controls were also run in each reaction. Relative quantification was calculated using the formula 2^−∆∆*C*t^. Normalization was performed with miR-191.

### Statistical analyses

Statistical analyses were performed with SPSS 22 (SPSS Inc, Chicago, IL, USA). In vitro experiments were repeated on at least three biological replicates. Data are presented as the mean ± SEM and analyzed using paired two-tailed Student’s *t* test, using Prism software version 7.0 (GraphPad software). Differences were considered significant where *p* ≤ 0.05.

## Results

### miR-328 targets SLC2A1

**Fig. 1:**
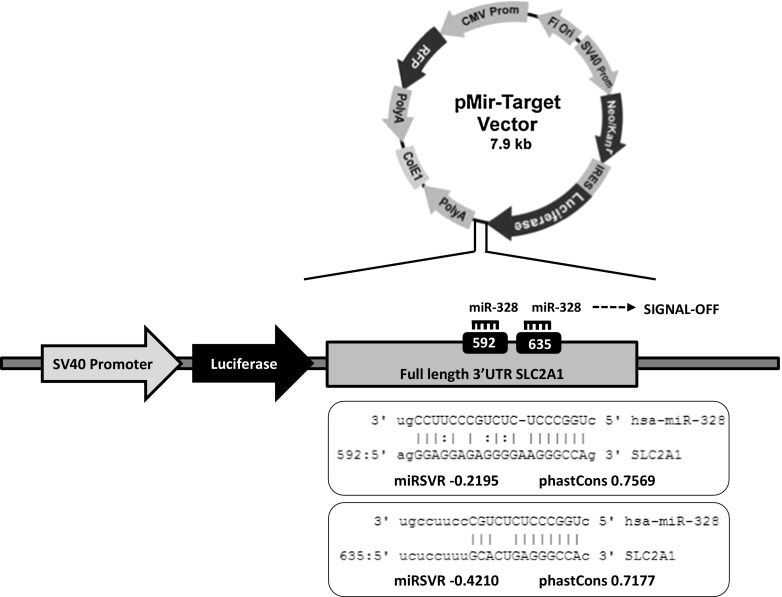
Luciferase construct was used to test whether miR-328 binds to SLC2A1. The 3′ UTRs of SLC2A1 were subcloned into the SV40-driven pMir-target luciferase vector (Origene)

Bioinformatic analysis suggested that SLC2A1 could be a potential target of miR-328. Tarbase showed that the inhibition of SLC2A1 in *Homo sapiens* is partially mediated by miR-328 in two target sites that are dependent on additional non-sequence-specific context features. These SLC2A1 target sites for hsa-miR-328 are located in the 592 and 635 nucleotide positions of the 5′ gene sequence. Their mirSVR scores are − 0.2195 and − 0.4210, respectively, indicating that miR-328 can potentially bind to its target gene SLC2A1, especially in the 635 position, which has a more negative mirSVR score. phastCons scores for the 592 and 635 nucleotide positions suggest that both show a high conservation rate across vertebrates (0.7569 and 0.7177, respectively) (Fig. 1).

### miR-328 directly regulates SLC2A1 in CC cells

**Fig. 2:**
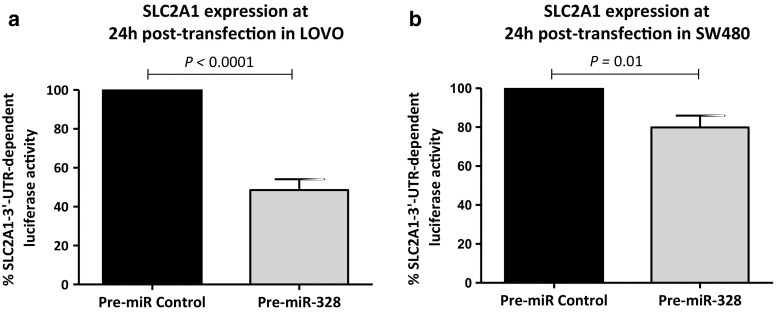
Determination of luciferase activities in **a** LOVO and **b** SW480 colon cancer cells transfected with luciferase expression constructs containing the SLC2A1 downstream of the luciferase gene on a SV40-driven vector. SLC2A1 is a direct target of miR-328. Luciferase activity of plasmid containing firefly luciferase associated with 3′ UTRs regions of SLC2A1 cotransfected with pre-miR-328 (100 nM) was reduced in **a** LOVO (*p* < 0.0001) and **b** SW480 (*p* = 0.01) cells. Cotransfection of SLC2A1 with a non-targeted pre-Negative Control did not cause a significant decrease in firefly luciferase activity. The values presented are the mean ± SEM of three independent experiments (paired two tailed Student’s *t* test)

To demonstrate the direct interactions between miR-328 and SLC2A1-3′UTR, a construct of this region was cloned into the pMir-Target plasmid downstream of a luciferase reporter gene (Fig. 1). A significant decrease was observed in luciferase activity in cells transfected with pMirTarget-SLC2A1 construct when treated with 100 nM pre-miR-328 relative to the same concentration of a pre-miR-Negative Control: 48.6% in LOVO (*p* < 0.0001) and 20.2% in SW480 (*p* = 0.01) (Fig. 2). There was no significant difference between cells transfected with the target constructs alone. This provides strong evidence of a direct molecular binding interaction between miR-328 and the 3′UTRs of SLC2A1, indicating that this gene could be a novel direct target of miR-328.

**Fig. 3:**
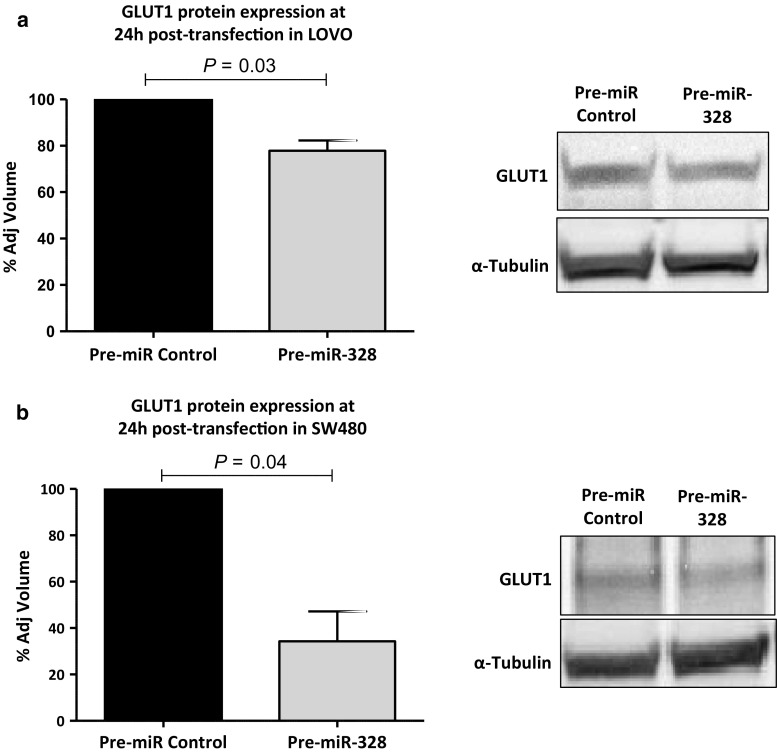
Western blot of **a** LOVO (*p* = 0.03) and **b** SW480 (*p* = 0.04) cells transfected with pre-Negative Control or pre-miR-328 and quantification of SLC2A1 signal relative to loading control α-tubulin

Western blot was carried out to determine if changes in miR-328 levels correlated with changes in SLC2A1-encoded protein levels, GLUT1. The results showed that protein levels of GLUT1 were reduced in cells transfected with pre-miR-328 compared to cells treated with the pre-miR-Negative Control: LOVO (*p* = 0.03); SW480 (*p* = 0.04) (Fig. 3). Taken together, these data indicate that miR-328 upregulation may contribute to the inhibition of GLUT1 in CC cells.

### miR-328 expression in patient samples

Forty-seven patients were included in the study. Median age was 73 years, and 29 (62%) were males. Thirty-three patients had stage I–II disease and 14 had stage III. Mean follow-up was 45.2 months (range 26.4–63.8) (Table 1).

**Table 1:** Patient characteristics

Characteristics	*N* (%)
Sex	
Male	29 (62)
Female	18 (38)
Median age	73
CEA levels	
≤ 5	31 (66)
> 5	16 (34)
C 19.9 levels	
≤ 37	43 (92)
> 37	4 (8)
Tumor location	
Left colon	23 (49)
Right colon	24 (51)
Tumor size (cm)	
≤ 5	34 (72)
> 5	12 (26)
Unknown	1 (2)
Histological type	
Well differentiated	42 (89)
Poorly differentiated	5 (11)
Preexistent polyp	
Absent	35 (75)
Present	12 (25)
Perilymphatic invasion	
Absent	44 (94)
Present	2 (4)
Unknown	1 (2)
TNM stage	
I–II	33 (70)
III	14 (30)
Adjuvant treatment	
Fluoropyrimidines	25 (53)
None	22 (47)
Relapsed	
Yes	14 (30)
No	33 (70)

*CEA* carcinoma embryonic antigen, *TNM* tumor, nodule, metastasis

**Fig. 4:**
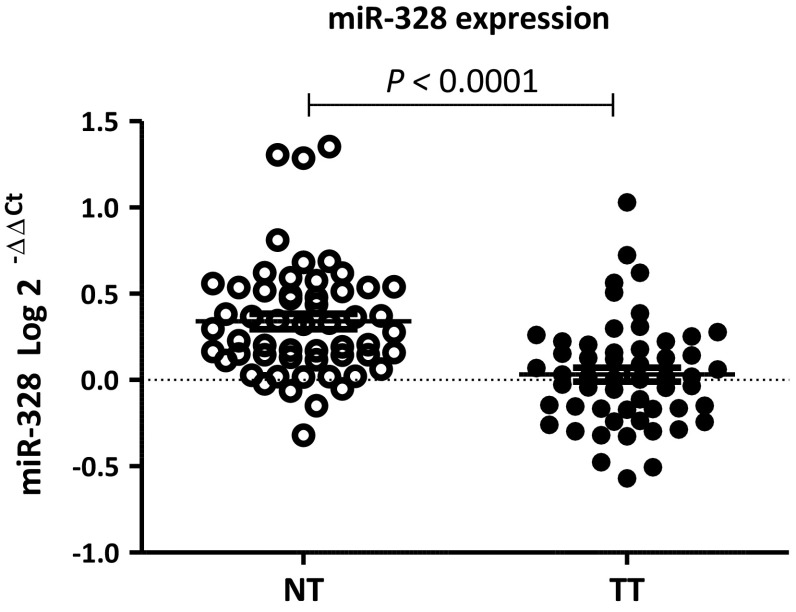
miR-328 expression in paired tumor and normal tissue samples from CC patients

Median miR-328 expression in tumor and normal tissue was − 0.3838 and − 0.0349, respectively. miR-328 was expressed at lower levels in tumor than in paired normal tissue (*p* < 0.0001) (Fig. 4).

## Discussion

In the recent years, accumulating evidence demonstrating the critical role of a large number of miRNAs as cancer regulators [25] indicate that it is crucial to understand the molecular mechanisms of miRNAs and their potential as therapeutic targets. To understand the potential effect of miR-328 on CC cells, we performed a target prediction analysis and luciferase reporter assays in CC cell lines treated with and without pre-miR-328. Our findings confirmed that miR-328 directly targets SLC2A1 in LOVO and SW480 cells, resulting in decreased levels of GLUT1 protein. Moreover, miR-328 expression was significantly lower in tumor than in normal tissue from CC patients. Tumor tissue is characterized by a high glucose uptake, mainly via GLUT1 [26], which is why a miR-328-mediated reduction of GLUT1, an important glucose transport protein, could compromise tumor survival.

Our results are in line with previous studies showing that miR-328 modulates both metabolic and non-metabolic pathways. miR-328 suppresses the survival of cancer cells by targeting PLCE1 in esophageal cancer [16], inhibits epithelial to-mesenchymal transition in renal tubular cells by targeting CD44 [17, 27], and regulates cell invasion and proliferation in glioma cells by targeting SFRP1 [18] and in melanoma cells by targeting TGFb2 [19]. In addition, miR-328 regulates the expression of breast cancer resistance protein (BCRP/ABCG2) in human cancer cells, resulting in increased chemosensitivity [20, 21]. In line with these results, a study focused on miR-328 and cancer stem cell properties suggested that miR-328 downregulation correlated with more invasive and tumorigenic phenotypes in vitro [28].

Recent studies have shown that some miRNAs secreted in the microenvironment by the tumor and niche cells could play an active role in modulating the pre-metastasic niche and subsequent metastatic progression. For example, in breast cancer, tumor-secreted miR-122 reprograms glucose metabolism in the pre-metastatic niche by inhibiting the glycolytic enzyme pyruvate kinase M2 (PKM2) [29], while in brain cancer, astrocyte-derived miR-19a secreted by the niche cells induces the loss of PTEN in tumor cells [30]. In both cases, the authors suggest that glucose is increased in the surrounding stroma. We can thus speculate that together with other accumulated metabolic molecules, the increased glucose represents a feed of nutrients and energy for the anabolic pathways required for the growth of the cancer cells when they arrive at the pre-metastatic niche.

Our findings indicate that changes in miR-328 levels correlate with changes in the levels of GLUT1, an SLC2A1-encoded protein, which has been confirmed by western blot analysis. As a result of this direct interaction between SLC2A1 and miR-328, when miR-328 levels are increased in cells transfected with its pre-miRNA, glucose uptake becomes reduced due to an inhibition of the glucose carrier GLUT1, leading to decreased glucose transport. Taken together with our previous findings that miR-328 expression in exosomes isolated from the tumor-draining vein of CC patients was associated with liver metastases [22] as well as previous studies reporting increased stromal glucose [29, 30], the results of the present study lead us to suggest that aberrant expression of miR-328 can modulate the metabolic environment in order to induce a pre-metastatic niche. Low levels of miR-328 in the tumor could allow an increase in GLUT1-mediated glucose uptake to support tumor growth while the miR-328 secreted by the cancer cells could inhibit the glucose uptake of distant stromal niche cells, resulting in stromal glucose accumulation and hence metastatic colonization.

In conclusion, to the best of our knowledge, our study is the first to demonstrate that miR-328 is potentially able to inhibit SLC2A1 and consequently regulate GLUT1-mediated glycolytic activity in cancer cells. Further exploration of the biological mechanisms underlying this interaction is warranted to evaluate the potential of miR-328 as a therapeutic target in CC.
